# Rituximab (monoclonal anti-CD20 antibody) induced posterior reversible encephalopathy syndrome (PRES): A case report and literature review

**DOI:** 10.1016/j.radcr.2024.11.070

**Published:** 2024-12-24

**Authors:** Praveen K. Sharma, Sanjaykanth Balachandar, Michael Antony Vikram, Pujitha Duvooru Sukumar

**Affiliations:** Department of Radio-Diagnosis, Saveetha Medical College and Hospital, Saveetha Nagar, Thandalam, Chennai, Tamil Nadu 602105, India

**Keywords:** Posterior leukoencephalopathy syndrome, Rituximab, Hypertension, Seizures, Anticonvulsants, Magnetic resonance imaging

## Abstract

Posterior reversible encephalopathy syndrome (PRES) is an uncommon neurological condition characterized by reversible subcortical vasogenic edema that primarily affects the posterior areas of the brain. Subcortical vasogenic edema resulting from endothelial injury and hypertension is the pathogenesis. Here, we present a 23-year-old female patient with systemic lupus erythematosus (SLE) and lupus nephritis who developed PRES following Rituximab (a monoclonal anti-CD-20 antibody) administration. The patient initially presented with severe headaches, visual disturbances, and an altered mental status. Neurological examination revealed bilateral cortical blindness, hyperreflexia, and seizures. Brain imaging, including MRI, demonstrated characteristic findings of PRES, with symmetric hyperintensities involving the occipital and parietal lobes on T2-weighted and FLAIR sequences, consistent with vasogenic edema. Rituximab is promptly discontinued, and the patient was managed with supportive care, including antiepileptic drugs and blood pressure control. Within days of Rituximab cessation, the patient showed gradual improvement in symptoms, with resolution of cortical blindness and normalization of MRI findings. Follow-up assessments revealed complete neurological recovery without residual deficits. This instance emphasizes how crucial it is to take into account PRES as a possible side effect in patients receiving Rituximab therapy, especially if those individuals have sudden neurological symptoms. The offending agent must be located and eliminated immediately for the best outcomes. Clinicians should maintain a high index of suspicion for PRES in patients receiving monoclonal anti-CD20 antibody therapies, immunosuppressants, and corticosteroids, facilitating timely diagnosis and intervention to prevent potentially life-threatening complications. More studies are necessary to clarify the pathophysiological mechanisms causing the PRES produced by Rituximab and to improve therapeutic approaches.

## Introduction

Reversible subcortical vasogenic edema, primarily affecting the posterior regions of the brain, is the hallmark of a rare but clinically significant neurological disorder known as posterior reversible encephalopathy syndrome (PRES). The symptoms of PRES, which were first identified by Hinchey et al. in 1996, usually include headaches, altered mental status, seizures, visual problems, and focal neurological impairments [1]. Although the precise pathophysiology of PRES remains incompletely understood, endothelial dysfunction leading to disrupted blood-brain barrier integrity and subsequent vasogenic edema formation is believed to play a central role [2]. While PRES has been associated with various predisposing factors such as hypertensive crises, eclampsia, autoimmune diseases, and immunosuppressive therapies, an increasing number of cases have been reported in association with monoclonal anti-CD20 antibody therapies, including Rituximab [3]. A chimeric monoclonal anti-CD20 antibody called Rituximab targets B cells' CD20 antigen. It is frequently used to treat various hematologic cancers and autoimmune diseases [4]. The mechanisms underlying Rituximab-induced PRES remain elusive; however, it is hypothesized that immune-mediated endothelial injury, cytokine release, and dysregulated cellular immune responses may contribute to developing PRES in susceptible individuals [5]. Despite its rarity, drug-induced PRES poses significant clinical challenges due to its potentially life-threatening complications and the necessity for prompt recognition and intervention. In this context, we present a case report of Rituximab-induced PRES in a patient with systemic lupus erythematosus (SLE) and lupus nephritis, highlighting the clinical features, diagnostic approach, management strategies, and potential mechanisms underlying this rare complication. Through this case report, we aim to enhance awareness among clinicians regarding the association between Rituximab therapy and PRES and emphasize the importance of vigilant monitoring and timely intervention in patients receiving monoclonal anti-CD20 antibody therapy.

## Case presentation

Presenting complaints: A 23-year-old female presented to the emergency department with complaints of a series of 4 seizures accompanied by severe headaches, visual disturbances, and altered mental status. Her mother observed the simultaneous upward rolling of both eyes and biting of the tongue.

Clinical history, examination, and initial treatment: On clinical history, a seizure-like episode was reported by her at home, and subsequently, she had a generalized tonic-clonic seizure in the emergency department, which ended with intravenous lorazepam. Her Glasgow Coma Scale (GCS) showed erratic values ranging from 5 to 9 (eyes score 2, verbal score 2, motor score 5). Her postictal phase lasted for 6 hours.

According to the patient's mother, the patient exhibited a moderate-intensity headache in the occipital region for 2 days before the first seizure. She observed a decline in vision for approximately 6 hours, leading up to the commencement of seizures. There was no evidence of a known seizure disorder in her medical history, and she had experienced no seizures before being admitted. She denied any history of hypertension, eclampsia, or autoimmune disorders. The patient's family history was unremarkable for neurological or autoimmune conditions.

Examining the patient revealed that they were listless and confused. The neurological assessment revealed bilateral cortical blindness, hyperreflexia, and intermittent generalized tonic-clonic seizures. Vital signs showed no signs of a hypertensive crisis and were within normal ranges. A complete blood count, an electrolyte panel, and renal function tests were among the laboratory procedures that yielded unremarkable results.

The patient has a documented history of Systemic Lupus Erythematosus (SLE) for 8 years, with the development of lupus nephritis 1 year ago. *Mycophenolate mofetil, Prednisolone and Tacrolimus* were given as the first line of treatment. Because of the lack of response to these medications, *Rituximab* was administered 5 days before the onset of the first seizure. Before being discharged from the hospital, the patient got a second dose. Throughout the treatment, no neurological symptoms were observed in the patient. After discharge, the patient displayed symptoms of visual blurring in both eyes and retro-orbital pain, with no documented loss of consciousness. The symptoms had developed acutely following the second dose of *Rituximab* therapy. Following this, she encountered 2 instances of seizures accompanied by a headache at her place of residence, prompting her immediate transfer to a healthcare institution where she received fosphenytoin via injection. Despite receiving treatment, she experienced another occurrence of a generalized tonic-clonic seizure.

**Fig. 1:**
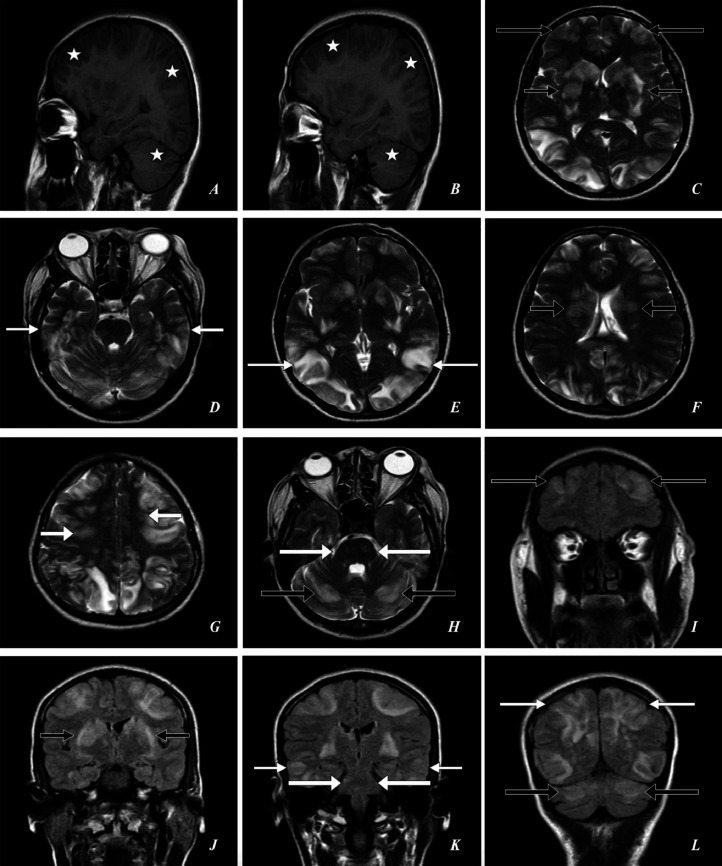
A 23-year-old female presented to the emergency department with complaints of a series of 4 seizures accompanied by severe headaches, visual disturbances, and altered mental status. *Emergency Magnetic Resonance Imaging (MRI)—Brain: (A) T1W Right Para-Sagittal, (B) T1W Left Para-Sagittal, (C, D, E, F, G, H) T2W Axial, (I, J, K, L) FLAIR Coronal* shows *T1W:* multifocal/multi-centric asymmetrical hypointensities in the deep gray matter, deep white matter, sub-cortical white matter U-fibers and cortical white matter *(white asterisks). T2W and FLAIR:* multifocal/multi-centric asymmetrical hyperintensities/areas (vasogenic edema) in the bilateral ganglio-capsular regions, bilateral thalami *(short thin black arrows),* bilateral frontal lobes *(large thin black arrows),* bilateral temporal lobes *(short thin white arrows),* bilateral parieto-occipital lobes *(large thin white arrows),* bilateral corona radiata *(short thick black arrows),* bilateral centrum semiovale *(short thick white arrows),* bilateral hemipons and bilateral middle cerebellar peduncles *(large thick white arrows),* bilateral cerebellar hemispheres *(large thick black arrows)* with effacement of adjacent cerebral sulci and cerebellar foliae.

**Fig. 2:**
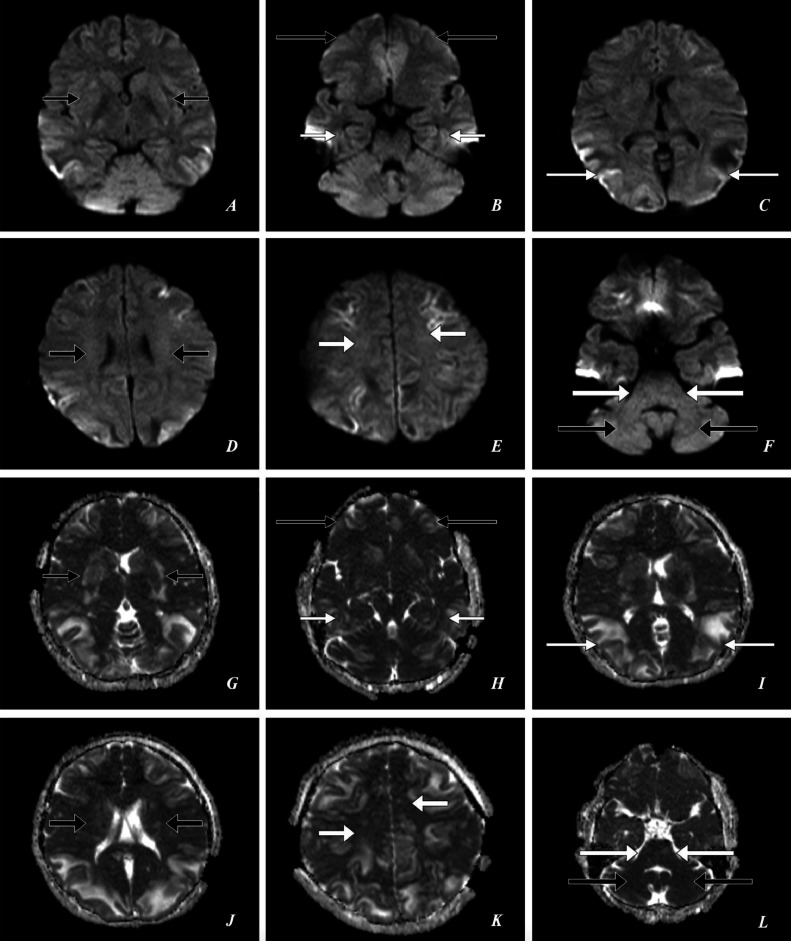
A 23-year-old female presented to the emergency department with complaints of a series of 4 seizures accompanied by severe headaches, visual disturbances, and altered mental status. *Emergency Magnetic Resonance Imaging (MRI)—Brain: (A, B, C, D, E, F) DWI Axial, (G, H, I, J, K, L) ADC Axial* shows *DWI:* few subtle areas of patchy reduced diffusivity and *ADC:* few areas of low signal intensities in the bilateral ganglio-capsular regions, bilateral thalami *(short thin black arrows),* bilateral frontal lobes *(large thin black arrows),* bilateral temporal lobes *(short thin white arrows),* bilateral parieto-occipital lobes *(large thin white arrows),* bilateral corona radiata *(short thick black arrows),* bilateral centrum semiovale *(short thick white arrows),* bilateral hemipons and bilateral middle cerebellar peduncles *(large thick white arrows),* bilateral cerebellar hemispheres *(large thick black arrows).*

**Fig. 3:**
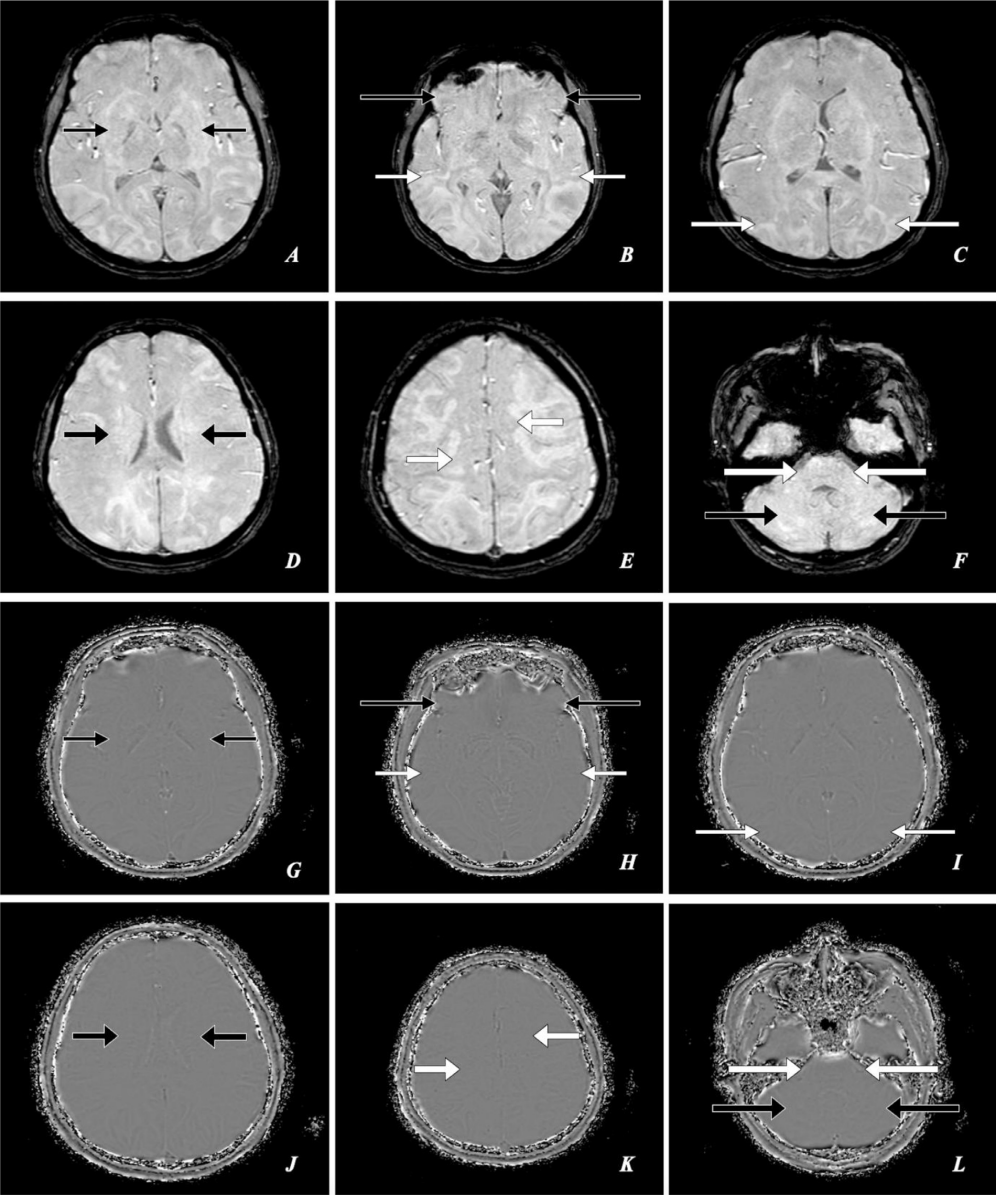
A 23-year-old female presented to the emergency department with complaints of a series of 4 seizures accompanied by severe headaches, visual disturbances, and altered mental status. *Emergency Magnetic Resonance Imaging (MRI)—Brain: (A, B, C, D, E, F) SWI Axial, (G, H, I, J, K, L) SWI-Phase Axial* shows *SWI:* no susceptibility artifacts and *SWI-Phase:* no susceptibility artifacts in the bilateral ganglio-capsular regions, bilateral thalami *(short thin black arrows),* bilateral frontal lobes *(large thin black arrows),* bilateral temporal lobes *(short thin white arrows),* bilateral parieto-occipital lobes *(large thin white arrows),* bilateral corona radiata *(short thick black arrows),* bilateral centrum semiovale *(short thick white arrows),* bilateral hemipons and bilateral middle cerebellar peduncles *(large thick white arrows),* bilateral cerebellar hemispheres *(large thick black arrows).*

**Fig. 4:**
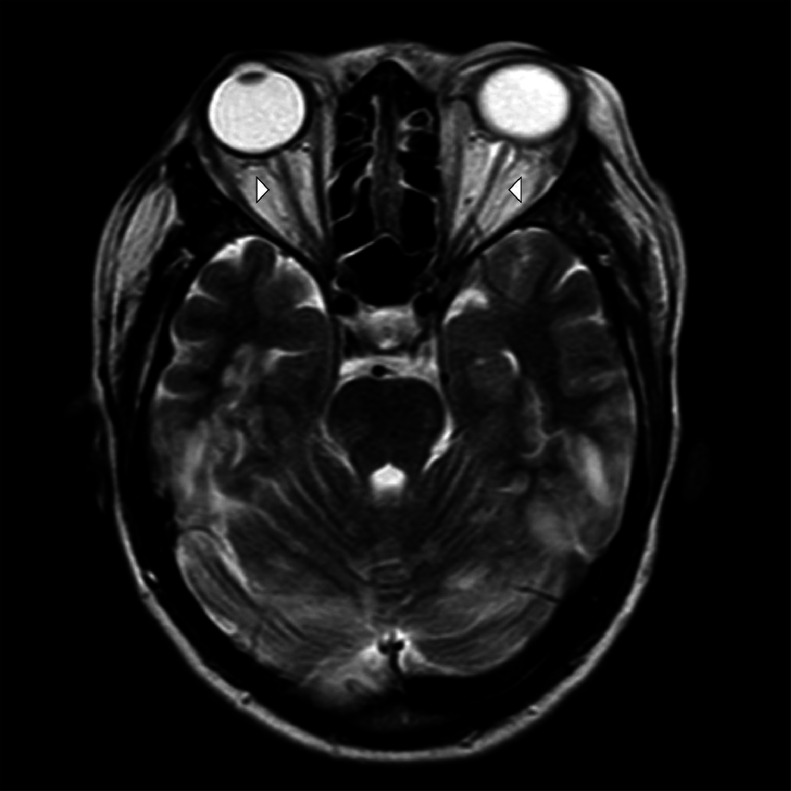
A 23-year-old female presented to the emergency department with complaints of a series of 4 seizures accompanied by severe headaches, visual disturbances, and altered mental status. *Emergency Magnetic Resonance Imaging (MRI)—Brain: T2W Axial* shows intra-orbital segment of bilateral optic nerves vertical moderate tortuosity with sub-arachnoid cistern prominent *— Intra-cranial hypertension (small arrow heads).*

Imaging findings: *Emergency magnetic resonance imaging (MRI) brain* Shows T1: multifocal/multi-centric asymmetrical hypointensities, and T2: hyperintensities/areas in the deep gray matter, deep white matter, sub-cortical white matter U-fibers and cortical white matter of bilateral cerebral hemispheres (bilateral ganglio-capsular region, bilateral thalami, bilateral frontal, parietal, temporal, occipital lobes, bilateral corona radiata, bilateral centrum semiovale) (predominantly in bilateral occipital lobes), bilateral cerebellar hemispheres with perifocal sub-cortical mild edema (likely vasogenic edema) with effacement of adjacent sulci (FLAIR: hyperintense, DWI: few areas of patchy reduced diffusivity, SWI: no susceptibility artifacts). T1: Few asymmetrical hypointensities, and T2: hyperintensities in the dorsal and ventral aspects of bilateral hemipons and bilateral middle cerebellar peduncles (FLAIR: hyperintense, DWI: no reduced diffusivity, SWI: no susceptibility artifacts) (Fig. 1, Fig. 2, Fig. 3, Fig. 4)**.**

Diagnosis: Based on clinical and imaging findings, the *Drug-induced [Rituximab (Monoclonal Anti-CD20 antibody) Posterior Reversible Encephalopathy Syndrome (PRES)* was considered.

Treatment: *Rituximab* therapy was promptly discontinued, and the patient was managed with supportive care, including antiepileptic drugs for seizure control and aggressive blood pressure management. Within days of *Rituximab* cessation, the patient showed gradual improvement in symptoms, with resolution of cortical blindness. The treatment for the edema involved the administration of furosemide injections, while the use of antiepileptic drugs, specifically levetiracetam injections, was maintained.

**Fig. 5:**
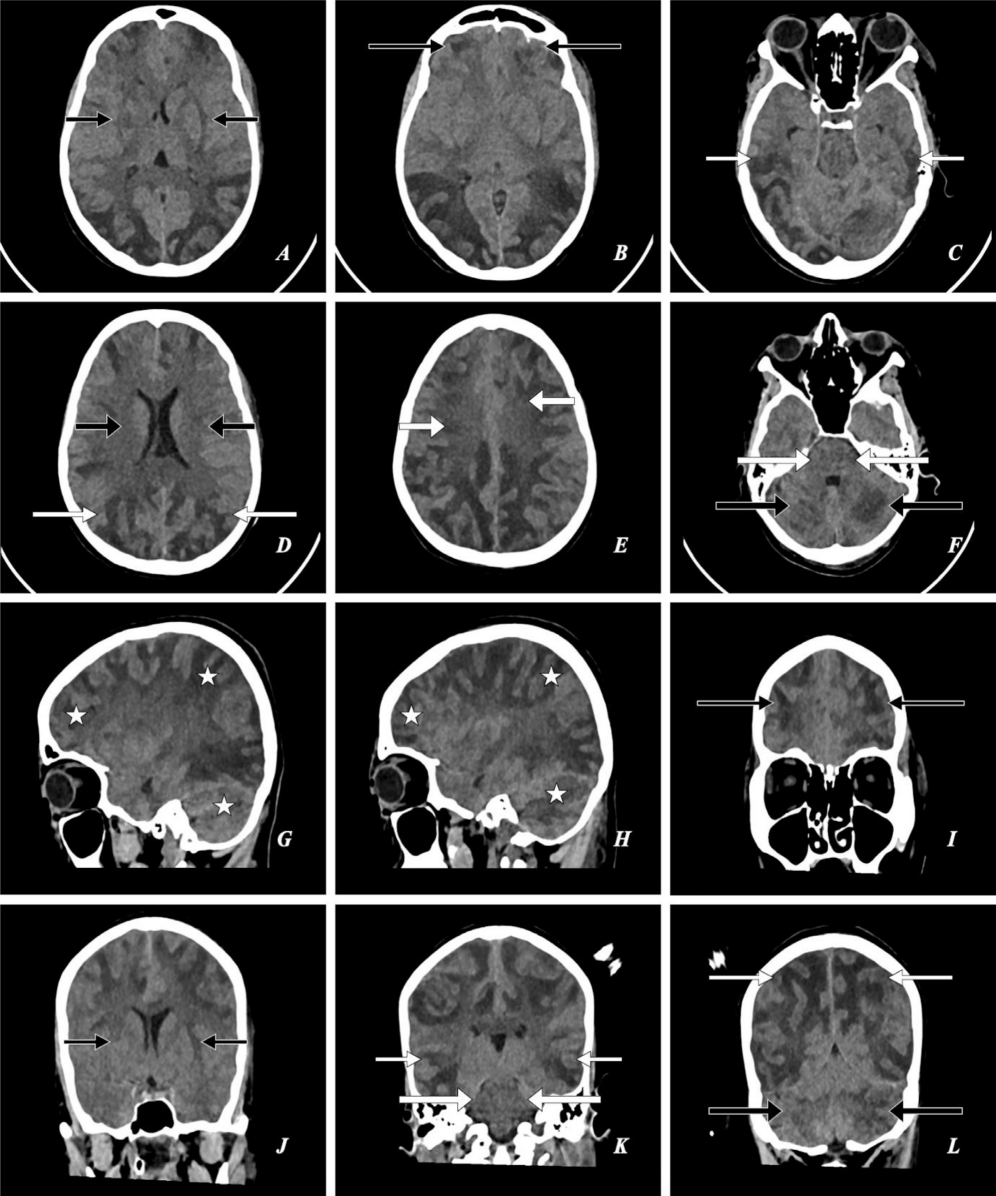
A 23-year-old female presented to the emergency department with complaints of a series of 4 seizures accompanied by severe headaches, visual disturbances, and altered mental status. *Follow-up (3 days) Computed Tomography (CT)—Brain: (A, B, C, D, E, F) Axial, (G) Right Para-Sagittal, (H) Left Para-Sagittal, (I, J, K, L) Coronal* shows multifocal/multi-centric asymmetrical hypodensities (vasogenic edema) in the bilateral ganglio-capsular regions, bilateral thalami *(short thin black arrows),* bilateral frontal lobes *(large thin black arrows),* bilateral temporal lobes *(short thin white arrows),* bilateral parieto-occipital lobes *(large thin white arrows),* bilateral corona radiata *(short thick black arrows),* bilateral centrum semiovale *(short thick white arrows),* bilateral hemipons and bilateral middle cerebellar peduncles *(large thick white arrows),* bilateral cerebellar hemispheres *(large thick black arrows).* Vasogenic edema in the deep gray matter, deep white matter, sub-cortical white matter U-fibers and cortical white matter *(white asterisks) [Significant reduction / resolution of Vasogenic edema].*

*Follow-up (3 days): Computed tomography (CT) of the brain* shows multifocal asymmetrical subtle hypodensities in the mentioned areas (near-complete resolution) (Fig. 5).

Follow-up: The patient's progress was monitored for 2 months. She remained asymptomatic and did not receive the drugs *Rituximab*. The patient is currently prescribed a nightly dose of the Hydroxychloroquine (200mg) tablet. The advice is to consume only 1.5 liters of fluids daily and restrict salt intake to less than 3 grams daily. The patient has been instructed to take a tablet of levetiracetam 500mg twice daily for neurological complications. During follow-up assessments, the patient demonstrated complete neurological recovery without residual deficits. Repeat imaging studies confirmed the resolution of vasogenic edema. The patient was subsequently counseled regarding the potential risks of *Rituximab* therapy and alternative treatment options for SLE.

This case highlights the importance of recognizing *Rituximab-induced Posterior Reversible Encephalopathy Syndrome (PRES)* as a rare but severe complication in patients receiving monoclonal anti-CD20 antibody therapy. Prompt cessation of the offending agent and supportive management are essential for favorable outcomes, with most patients demonstrating complete neurological recovery.

## Discussion

Incidence and Demographics: An uncommon neurological condition known as posterior reversible encephalopathy syndrome (PRES) is characterized by reversible subcortical vasogenic edema that primarily affects the posterior areas of the brain. The incidence of PRES has been estimated to be around 0.01% of all hospital admissions, but it may be underreported due to its variable clinical presentation and lack of recognition. Although PRES can afflict people of any age, it is more frequently seen in adults, especially those between the ages of 20 and 50. However, cases have been reported in pediatric populations as well. The demographic distribution of PRES reflects its association with various predisposing factors, including hypertension, renal disease, autoimmune disorders, eclampsia, and immunosuppressive therapies, among others [6].

Age of Presentation: PRES can present across a wide age range, with reported cases ranging from children as young as a few months old to elderly individuals. However, adults—especially those in their middle years—tend to experience it more frequently. The age at presentation may vary depending on the underlying predisposing factors and comorbidities present in the patient population [7].

Pathophysiology and Causes: The etiology of posterior-segmental brain edema (PRES) is characterized by dysregulation of cerebral autoregulation, endothelial dysfunction, and blood-brain barrier disruption. These factors culminate in vasogenic edema. The exact mechanisms underlying PRES are not fully understood, but they are believed to involve an imbalance between cerebral perfusion and vascular integrity. Various factors have been implicated in the development of PRES, including hypertension, renal impairment, autoimmune diseases, eclampsia, immunosuppressive therapies, and cytotoxic drugs. These factors can disrupt cerebral autoregulation and endothelial function, leading to increased vascular permeability and the development of vasogenic edema. Monoclonal anti-CD20 antibody therapy, such as Rituximab, have also been associated with PRES, although the precise mechanisms remain unclear. Rituximab-induced PRES is thought to result from immune-mediated endothelial injury, cytokine release, and dysregulated cellular immune responses triggered by monoclonal anti-CD20 antibody therapy [8].

Clinical Features: The clinical presentation of PRES typically includes the acute onset of symptoms such as severe headaches, altered mental status, seizures, visual disturbances, and focal neurological deficits. Patients may show different degrees of damage to their consciousness, from disorientation to unconsciousness. The diagnosis can be difficult because the clinical signs and symptoms can be vague and similar to those of other neurological disorders. However, a thorough clinical assessment combined with imaging studies is essential for accurate diagnosis and timely management [9].

Associated Abnormalities: PRES can be associated with various predisposing factors, including hypertension, eclampsia, autoimmune diseases, renal disorders, and *Rituximab (Monoclonal anti-CD20 antibody).* Patients with underlying conditions that disrupt cerebral autoregulation or compromise blood-brain barrier integrity may be at an increased risk of developing PRES. Additionally, certain medications and chemotherapy agents have been implicated in PRES, highlighting the diverse etiologies associated with this syndrome [10].

Location: PRES predominantly affects the posterior regions of the brain, including the parietooccipital lobes. However, other brain regions, including the frontal and temporal lobes, cerebellum, and brainstem, can also be involved. In imaging studies, the characteristic distribution of vasogenic edema helps distinguish PRES from other neurological conditions affecting similar brain regions [11].

Imaging Findings: Characteristic imaging findings on magnetic resonance imaging (MRI) include symmetric hyperintensities on T2-weighted and fluid-attenuated inversion recovery (FLAIR) sequences, indicative of vasogenic edema in the affected brain regions. These abnormalities typically involve the subcortical white matter and may spare the overlying cortex, contributing to the reversible nature of the syndrome. In severe cases, diffusion-weighted imaging (DWI) may demonstrate restricted diffusion, reflecting cytotoxic edema and neuronal injury [12].

Treatments: Management of Rituximab-induced PRES involves prompt discontinuation of Rituximab therapy and supportive care. Treatment must include strong blood pressure management and antiepileptic medication for seizure control. Close monitoring of neurological status, vital signs, and laboratory parameters is warranted to assess therapy response and detect potential complications. In most cases, patients show gradual improvement with cessation of the offending agent and supportive measures, resolution of symptoms, and normalization of imaging findings over time [13].

Differential Diagnosis: The differential diagnosis for PRES includes various neurological conditions that can present with similar clinical features and imaging findings. Among the illnesses to be taken into account include:

• Ischemic stroke.
• Infections of the central nervous system.
• Cerebral bleeding.
• Demyelinating diseases (like multiple sclerosis).

A thorough evaluation, including clinical history, physical examination, laboratory tests, and imaging studies, is essential for accurate diagnosis and appropriate management. Response to treatment and the evolution of clinical and radiological findings over time can help differentiate PRES from other conditions [14].

## Conclusion

*Rituximab-induced Posterior Reversible Encephalopathy Syndrome (PRES)* is a rare but potentially serious complication associated with monoclonal anti-CD20 antibody therapy, particularly in patients with underlying hematologic malignancies or autoimmune diseases. The etiology of posterior-segmental brain edema (PRES) is characterized by dysregulation of cerebral autoregulation, endothelial dysfunction, and blood-brain barrier disruption. These factors culminate in vasogenic edema. Prompt recognition and management are essential for favorable outcomes, with most patients demonstrating gradual improvement with cessation of the offending agent and supportive care. Despite its rarity, clinicians should maintain a high index of suspicion for PRES in patients receiving Rituximab or other monoclonal antibody therapies, facilitating timely diagnosis and intervention to prevent potentially life-threatening complications. More study is required to understand the underlying mechanisms and develop treatment plans for this uncommon neurological condition.

## Literature reviews

Few studies and case reports have contributed to our understanding of drug-induced Posterior Reversible Encephalopathy Syndrome (PRES), including cases associated with Rituximab. Here, we provide a summary of key findings from relevant literature:

1. Rituximab-induced PRES: Studies by Khosroshahi et al. [15] have reported cases of PRES associated with Rituximab therapy in patients with autoimmune diseases and hematologic malignancies. These cases highlight the importance of recognizing PRES as a potential complication of Rituximab treatment and prompt discontinuation of therapy in affected patients. Rituximab-induced PRES is thought to result from immune-mediated endothelial injury and dysregulated cellular immune responses triggered by the monoclonal anti-CD20 antibody therapy.
2. Pathophysiology and Mechanisms: Research by Fugate et al. [16] elucidated the underlying mechanisms of PRES, emphasizing the role of endothelial dysfunction and disrupted cerebral autoregulation. Drug such as Rituximab may contribute to PRES through various mechanisms, including immune-mediated endothelial injury, alterations in vascular tone, and disruption of the blood-brain barrier.
3. Clinical Features and Diagnosis: Faille et al. [17] discuss the clinical spectrum of PRES, emphasizing the diverse presentations ranging from headaches to seizures and visual disturbances.
4. Imaging Findings: Nakamura et al. [18] discuss the imaging characteristics of PRES are extensively reviewed by various authors, including (2018), who discuss the typical MRI findings of vasogenic edema in the posterior regions of the brain.
5. Treatment Strategies: Shieh et al. [19] discuss the management of PRES, emphasizing the role of supportive care, including seizure control and blood pressure management.
6. Outcomes and Prognosis: McKinney et al. [20] explore the long-term outcomes and prognosis of PRES and discuss the reversibility of symptoms and imaging findings with appropriate management.
7. Risk Factors and Predisposing Conditions: Ando et al. [21] describes the role of predisposing factors such as hypertension, renal impairment, autoimmune diseases, and immunosuppressive therapies in the development of PRES.

Literature reviews provide valuable insights into the epidemiology, pathophysiology, clinical features, management, and outcomes of Rituximab-induced PRES, guiding clinicians in the recognition and optimal management of this rare neurological complication.

## Limitations

While the case report provides valuable insights into drug-induced Posterior Reversible Encephalopathy Syndrome (PRES) associated with Rituximab, there are several limitations that should be acknowledged:

1. Single Case Report: The study is based on a single case report, which limits the generalizability of findings to a broader population. The presentation, course, and outcomes observed in this case may not be representative of all cases of drug-induced PRES.
2. Causality Assessment: Establishing a direct causal relationship between the implicated drugs (Rituximab) and the development of PRES can be challenging. Other factors, such as underlying comorbidities or concurrent medications, may have contributed to the observed neurological symptoms.
3. Confounding Variables: The presence of confounding variables, such as concomitant use of multiple medications or underlying medical conditions, can complicate the interpretation of findings. Without a control group or systematic assessment of potential confounders, it is difficult to isolate the effects of individual drugs on PRES development.
4. Publication Bias: There may be a bias towards reporting cases with unusual or severe presentations, leading to an overrepresentation of certain drug associations with PRES in the literature. This bias can affect the perception of the true incidence and risk profile of drug-induced PRES.
5. Limited Follow-up: The duration of follow-up in the case report may be insufficient to fully capture the long-term outcomes and prognosis of drug-induced PRES. Complications or recurrence of symptoms may occur beyond the timeframe reported in this study.
6. Generalizability: The patient characteristics, underlying medical conditions, and treatment regimens described in the case report may not be applicable to all clinical settings. Variability in patient demographics, drug dosages, and treatment protocols can influence the risk of PRES development.
7. Reporting Bias: There may be limitations in the completeness and accuracy of data reported in the case study, including missing information or subjective interpretation of clinical findings. Inadequate documentation or retrospective data collection can introduce reporting bias and affect the reliability of study findings.
8. Ethical Considerations: Patient privacy and confidentiality must be maintained in the reporting of case details. Certain clinical information may be omitted or generalized to protect the identity of the patient, which could impact the comprehensiveness of the case report.

Addressing these limitations and conducting further research, such as larger case series or prospective studies, is essential for better understanding the epidemiology, pathophysiology, and management of drug-induced PRES. Collaborative efforts across multiple institutions and rigorous methodology can help overcome these challenges and provide more robust evidence on the association between specific drugs and PRES development.

## Patient consent

A written and informed consent was obtained from the patient for publication of this case report.
